# Evaluation of Different Controllers for Sensing-Based Movement Intention Estimation and Safe Tracking in a Simulated LSTM Network-Based Elbow Exoskeleton Robot

**DOI:** 10.3390/s26020387

**Published:** 2026-01-07

**Authors:** Farshad Shakeriaski, Masoud Mohammadian

**Affiliations:** Faculty of Science and Technology, University of Canberra, Canberra, ACT 2617, Australia; masoud.mohammadian@canberra.edu.au

**Keywords:** upper limb assistive exoskeleton robot elbow orthosis, deep learning, PID control, sliding mode control, impedance control

## Abstract

Control of elbow exoskeletons using muscular signals, although promising for the rehabilitation of millions of patients, has not yet been widely commercialized due to challenges in real-time intention estimation and management of dynamic uncertainties. From a practical perspective, millions of patients with stroke, spinal cord injury, or neuromuscular disorders annually require active rehabilitation, and elbow exoskeletons with precise and safe motion intention tracking capabilities can restore functional independence, reduce muscle atrophy, and lower treatment costs. In this research, an intelligent control framework was developed for an elbow joint exoskeleton, designed with the aim of precise and safe real-time tracking of the user’s motion intention. The proposed framework consists of two main stages: (a) real-time estimation of desired joint angle (as a proxy for movement intention) from High-Density Surface Electromyography (HD-sEMG) signals using an LSTM network and (b) implementation and comparison of three PID, impedance, and sliding mode controllers. A public EMG dataset including signals from 12 healthy individuals in four isometric tasks (flexion, extension, pronation, supination) and three effort levels (10, 30, 50 percent MVC) is utilized. After comprehensive preprocessing (Butterworth filter, 50 Hz notch, removal of faulty channels) and extraction of 13 time-domain features with 99 percent overlapping windows, the LSTM network with optimal architecture (128 units, Dropout, batch normalization) is trained. The model attained an RMSE of 0.630 Nm, R^2^ of 0.965, and a Pearson correlation of 0.985 for the full dataset, indicating a 47% improvement in R^2^ relative to traditional statistical approaches, where EMG is converted to desired angle via joint stiffness. An assessment of 12 motion–effort combinations reveals that the sliding mode controller consistently surpassed the alternatives, achieving the minimal tracking errors (average RMSE = 0.21 Nm, R^2^ ≈ 0.96) and showing superior resilience across all tasks and effort levels. The impedance controller demonstrates superior performance in flexion/extension (average RMSE ≈ 0.22 Nm, R^2^ > 0.94) but experiences moderate deterioration in pronation/supination under increased loads, while the classical PID controller shows significant errors (RMSE reaching 17.24 Nm, negative R^2^ in multiple scenarios) and so it is inappropriate for direct myoelectric control. The proposed LSTM–sliding mode hybrid architecture shows exceptional accuracy, robustness, and transparency in real-time intention monitoring, demonstrating promising performance in offline simulation, with potential for real-time clinical applications pending hardware validation for advanced upper-limb exoskeletons in neurorehabilitation and assistive applications.

## 1. Introduction

Upper limb robotic exoskeletons, practical systems in neuromuscular rehabilitation, are intended to aid patients with motor deficits including stroke, spinal cord injury, Parkinson’s disease, or post-surgical muscular weakness [1,2]. These technologies aim to restore motor autonomy, reduce muscular tension, prevent atrophy, and accelerate the rehabilitation process [3]. Unlike passive prostheses, active exoskeletons provide assistive force that aligns with the user’s mobility objectives using electric or hydraulic actuators [4,5]. This capability improves natural human–robot interaction and optimizes the user experience to better conform to typical biomechanical behavior [6]. Attaining precise, reliable, and responsive control in real-world settings requires tackling complex challenges such as signal delay, sensor interference, and variations in system dynamics [7]. A crucial element of exoskeleton control is the accurate detection of the user’s movement intentions [8]. Electromyography (EMG) data provide non-invasive and direct assessments of muscle activity, providing significant insights into the degree and pattern of contraction [9]. Progress in electrode array technology enables precise recording of the spatial distribution of muscle activation using high-density HD-sEMG data [10,11]. This data offers superior insights into inter-muscular coordination and real-time fluctuations in contraction patterns compared to traditional EMG using a limited number of electrodes [12]. Nonetheless, the analysis of these high-volume and chaotic inputs requires complex preprocessing methods, feature extraction, and nonlinear modeling to accurately predict joint torque or angle in real time [13].

Early research in upper-limb exoskeleton control primarily focused on methods based on electromyography (EMG) signals [14]. Dennis introduced one of the EMG-driven control systems for an elbow–shoulder exoskeleton, which estimated joint torque using time-domain features such as RMS and MAV and employed a PID controller with fixed gains for tracking [15]. This approach was simple to implement but exhibited high tracking error (RMSE > 5 Nm) in the presence of signal noise and varying effort levels. Falzarano proposed impedance control for an elbow exoskeleton that modeled spring-damper behavior to increase interactive compliance but relied on manual tuning of parameters and performed poorly in rotational movements (pronation/supination) due to variable muscle coordination and signal noise in sEMG [16]. Classical PID controllers, while simple, exhibit high sensitivity to EMG noise and nonlinear muscle dynamics, resulting in large tracking errors (typically RMSE > 5–10 Nm) and instability across effort levels [15]. In one study, a 3-DOF exoskeleton robot for shoulder rehabilitation was designed, utilizing SMC to track optimized trajectories, offering advantages such as low weight, an open circular mechanism for the third joint to address issues with long wiring and closed mechanisms, allowance for shoulder translational DOFs, ease of use, patient comfort, and effective rejection of disturbances like patient hand tremor; however, the inherent nonlinearity of rehabilitation robots and the need for nonlinear controllers to cope with uncertainties and parameter variations were highlighted as key challenges [17]. In another work, an adaptive fast terminal sliding mode controller combining fuzzy logic and PID was proposed for a pneumatic artificial muscle (PAM)-actuated robotic arm assisting elbow joint rehabilitation, achieving steady-state tracking errors less than 5 degrees and overshoot-free responses; nevertheless, the time-varying nonlinear dynamics, uncertain modeling, and hysteresis characteristics of PAM actuators necessitate online compensators and pose significant control difficulties [18]. Overall, the effectiveness of SMC in enhancing the performance of elbow exoskeleton robots is demonstrated, but persistent issues such as model uncertainties, actuator hysteresis, and patient-induced disturbances call for more advanced adaptive control strategies.

Recent years have seen rapid progress in deep learning for EMG-based intention estimation, including convolutional neural networks (CNNs) for spatial feature extraction [19,20,21,22], deep Q-networks (DQN) for decision-making in assistive control, and hybrid methods such as feature cross-layer interaction approaches (FCIHMRT) for improved generalization [23,24,25]. These methods show promise in complex multi-class gesture recognition or adaptive control. However, for real-time regression of continuous joint intention from streaming HD-sEMG in a control loop, recurrent architectures like remain highly suitable due to their effectiveness in modeling temporal dependencies, low computational overhead, and strong performance on sequential physiological signals with limited training data [26,27,28]. The present work thus employs an optimized LSTM for intention estimation while focusing on comprehensive comparison of classical controllers (PID, impedance, sliding mode) in the resulting hybrid framework.

Subsequent advancements have explored innovative designs and actuation methods to address these limitations. For instance, the Elbow-sideWINDER exoskeleton [29] introduces a wearable device for industrial ergonomics, focusing on minimizing joint misalignment discomfort through kinematics estimation, load compensation, and friction control, demonstrating significant reductions in muscle activation (up to 38.8% for biceps brachii) during load-lifting tasks. Similarly, a 2DOF exoskeleton for elbow and forearm rehabilitation [30] employs nonlinear sliding mode control for trajectory tracking in passive exercises, achieving effective naturalistic movements but highlighting challenges in dynamic environments. The NEUROExos system [31] incorporates a passive alignment mechanism and variable impedance actuation, enabling both robot-in-charge and patient-in-charge modes with adjustable stiffness (24–56 N·m/rad), though it requires careful calibration for user variability. Other works, such as an EMG-fused fuzzy neuro control for 1DOF elbow support [32], adapt impedance based on skin surface EMG and wrist force, improving flexibility for vague biological signals but facing issues with signal noise. Actuation innovations include shape memory alloy (SMA)-based exoskeletons [33,34], which offer lightweight (<1 kg), noiseless operation for flexion–extension and pronation–supination, suitable for evaluation and therapy, albeit with slower response times compared to traditional actuators. Finally, a master–slave system using modified computed torque control [35] facilitates remote rehabilitation, showing efficient trajectory following but underscoring the need for robust integration of HD-sEMG for real-time applications. With advancements in sensor technology, the use of HD-sEMG signals gained attention. Rojas-Martínez and colleagues published a public HD-sEMG dataset from 12 healthy individuals across four isometric tasks (flexion, extension, pronation, supination) and three effort levels (10, 30, 50 percent MVC), which has become a standard reference for future studies [36].

In this research, an intelligent hybrid control framework has been developed for an elbow joint robot, based on the integration of a deep learning LSTM network for real-time desired joint angle estimation from HD-sEMG features. This framework is designed to achieve precise and safe trajectory tracking in the presence of noise, nonlinear muscle behavior, parametric variations, and external disturbances, filling the gap in real-time hybrid frameworks by integrating an HD-sEMG-trained LSTM and three controllers, providing comprehensive evaluation across 12 motion–effort combinations. Three controllers—PID, impedance, and sliding mode—are implemented separately and compared afterward.

## 2. Materials and Methods

### 2.1. Overview

**Figure 1:**
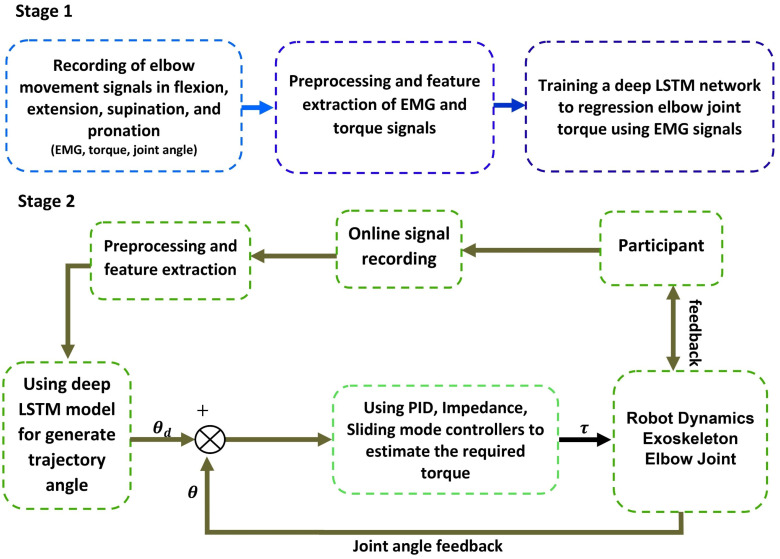
Overview of the proposed framework: (**Stage 1**) offline training stage of the LSTM model for estimation of desired elbow joint angle; (**Stage 2**) online real-time hybrid controller implementation stage.

In this research, an intelligent control framework is designed and implemented for parameter estimation of an elbow joint robot controller, developed with the aim of precise and safe real-time tracking of the user’s motion intention. The proposed methodology is organized into two main stages, schematically illustrated in Figure 1. In the first stage, the public HD-sEMG dataset published by Rojas-Martínez and colleagues [36] is used, which includes high-density surface electromyography signals (recorded with two-dimensional arrays and 10 mm electrode spacing) from 12 healthy males during isometric contractions in four motor tasks (flexion, extension, pronation, supination) and three effort levels (10%, 30%, 50% MVC). EMG signal preprocessing includes bandpass filtering at 12–350 Hz (4th-order Butterworth, bidirectional), removal of 50 Hz power line interference using a notch filter, and detection and replacement of faulty channels (≈6%) via neighborhood mean interpolation based on all data. Thirteen time-domain features are extracted for each of the three recorded channels from the signals of five key muscles (biceps brachii, triceps brachii, anconeus, brachioradialis, pronator teres), including RMS, MAV, VAR, etc. A multilayer LSTM network with optimized architecture is trained to real-time estimate the elbow desired joint angle from the EMG feature vector (${\hat{\theta }}_{desired}=f_{\mathrm{LSTM}}(x_{\mathrm{EMG}})$) In the second stage, the trained LSTM model is integrated in real time into the exoskeleton control loop, forming an online real-time hybrid control system. Live EMG signals are received from the user, preprocessed and time-domain-featured, and fed into the LSTM model to generate outputs in real time. Three controllers—PID, impedance, and sliding mode—are tested to produce the control angle (θ) based on the real-time error ($e=\theta_{desired}-\theta$).

### 2.2. Dataset

The public and validated high-density surface electromyography (HD-sEMG) signal dataset of isometric contractions of elbow muscles in healthy individuals is used in this research. The dataset consists exclusively of 12 healthy male volunteers (mean age 28.3 ± 5.5 years, height 177.8 ± 6.0 cm, weight 75.7 ± 8.7 kg), limiting direct evaluation of gender- and age-related differences in muscle activation patterns or EMG signal characteristics [36]. While this provides a controlled benchmark for initial algorithm development, generalization to female subjects, older adults, or clinical populations remains to be verified. The dataset was designed to investigate spatial distribution patterns of sEMG signals over upper limb muscles during voluntary isometric contractions. Signals were recorded from five key upper limb muscles (biceps brachii, triceps brachii, anconeus, brachioradialis, and pronator teres) in the dominant hand (right in all cases). Four different isometric tasks involving pronation/supination and flexion/extension related to forearm movements were performed. Each task was recorded at three effort levels (10%, 30%, and 50% of maximum voluntary contraction (MVC)) for 10 s. For signal recording, three two-dimensional electrode arrays (with gel-filled silver electrodes, 5 mm diameter, and 10 mm inter-electrode distance) were used. Array 1 was placed on the forearm covering the anconeus, pronator teres, and brachioradialis muscles (approximately 120 channels in a large grid configuration); Array 2 on the distal arm covering the biceps brachii (120 channels); and Array 3 on the proximal arm covering the triceps brachii (120 channels) [36]. Signals were sampled at 2048 Hz, with a bandwidth of 10–750 Hz, using OT Bioelettronica amplifiers (CMRR > 90 dB and input impedance > 300 MΩ at 50 Hz). A driven right leg (DRL) circuit and virtual ground were used to reduce common-mode interference. Additionally, torque signals from two torque sensors (range 150 Nm) were recorded to provide visual feedback to participants. Participants were positioned in a standard posture: sitting upright, arm parallel to the sagittal plane, elbow at 45 degrees, shoulder abducted at 90 degrees, and forearm rotated to 90 degrees (thumb up) [36].

### 2.3. Preprocessing Analysis

**Figure 2:**
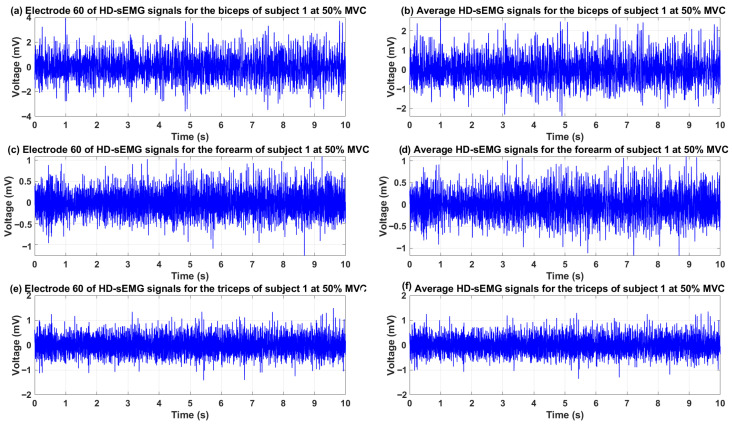
Examples of representative averaged HD-sEMG signals after preprocessing and channel averaging. Shown are signals from the three arrays (biceps brachii, triceps brachii, forearm muscles) during an isometric elbow flexion task at 50% MVC for one representative subject.

In this research, preprocessing of HD-sEMG signals and torque data is performed in a fully automated and uniform manner for all 12 subjects, 4 motor tasks (flexion, extension, pronation, supination), and 3 effort levels (10%, 30%, 50% MVC). The process is organized into two main stages: filtering, channel averaging, and time-domain feature extraction, followed by feature aggregation and formation of the final dataset. Raw HD-sEMG data (from 3 electrode arrays) and torque for each subject–task–effort combination are loaded from specified paths. For each channel, high-pass (cutoff frequency 5 Hz), low-pass (cutoff frequency 500 Hz), and band-stop (49–51 Hz band) filters are applied. The band-stop filter is a 4th-order Butterworth and bidirectional (zero-phase). After filtering, faulty channels (≈6%) are detected and replaced via neighborhood mean interpolation. The use of HD-sEMG arrays (with multiple small electrodes per muscle region) provides key advantages over traditional sparse or larger electrodes: higher signal-to-noise ratio (SNR) due to spatial averaging of closely spaced channels, robustness to electrode shift or skin variations, and effective handling of faulty channels through interpolation—features that enhance reliability in real-world applications [36]. Subsequently, the average of valid channels in each array is computed to obtain a single representative signal per muscle group (biceps brachii, triceps brachii, and forearm muscles). These averaging yields three high-quality signals while preserving global muscle activation patterns, reducing noise, and mitigating local artifacts that could bias feature extraction if only a few channels were selected. Figure 2 shows an example of representative averaged HD-sEMG signals after preprocessing and channel averaging. In comparison, using fewer channels with larger electrodes (e.g., two per array) would introduce greater spatial filtering, lower SNR, and reduced tolerance to misalignment or faults. From the filtered signal of each muscle, 13 standard time-domain features are extracted in overlapping windows. The window length is 100 samples (≈48.8 ms) and overlap is 99 samples (99%). Features are scaled to the [0,1] interval using min-max normalization. The formulas for calculating the features are presented in Table 1.

In all these formulas, $x_{i}(n)$ is the signal value at sample i of window n with length W. The moment signal was smoothed with a moving average with a window length of 50 samples (≈24.4 milliseconds). For each array, a 39 × N feature matrix was formed. The preprocessed matrices effectively removed noise, preserved temporal continuity, and provided a high-quality dataset for training the LSTM model. For a more precise analysis of the mechanical responses of the elbow joint and its correlation with muscle activation patterns, the measured torque data were converted to torsional angle values presented as follows:

(1) $$\theta =\frac{T}{k_{\mathrm{joint}}}$$

where θ represents the torsional angle in radians, T is the applied torque in Newton-meters, and $k_{\mathrm{joint}}$ is the torsional stiffness of the elbow joint in Nm/rad. Due to biomechanical differences between different elbow movements, torsional stiffness values were considered separately for each type of movement, as stated in Table 2 based on valid biomechanical studies reporting joint stiffness and torque-discomfort profiles during isometric tasks [44,45]. These studies provided data on forearm torque strengths and discomfort under varying elbow angles, forearm rotations, and effort levels, from which approximate stiffness values were derived to reflect physiological behavior. The obtained angular values were compared with similar data in biomechanical literature, confirming that the ranges fell within established physiological norms: typically, 100–146° for functional/practical flexion–extension arcs and 100–170° total (50–85° each direction) for pronation–supination rotation. Since the main relationship provides the torsional angle in radians, while the angle in degrees is required, the conversion relationship from radians to degrees is as follows:

(2) $$\theta_{\mathrm{degree}}=\theta_{\mathrm{radian}}\times \frac{180}{\pi }$$

By substituting relation 1 into conversion Formula (2), the final formula for calculating the torsional angle in degrees is obtained:

(3) $$\theta_{\mathrm{degree}}=\frac{T}{k_{\mathrm{joint}}}\times \frac{180}{\pi }$$

Since the dataset involves isometric contractions at a fixed elbow posture [36], the measured torque directly reflects muscle effort and movement intention rather than actual joint displacement. The conversion to angle using task- and effort-specific joint stiffness (Table 2) provides an approximation of the desired or intended joint angle that would result if the joint were unconstrained. This proxy is physiologically meaningful, as EMG amplitude strongly correlates with generated muscle torque during isometric tasks, and torque relates to potential angular displacement via joint compliance. The resulting angular values align with functional elbow ranges reported in the biomechanical literature (e.g., 30–130° for practical flexion–extension and ±50° for pronation–supination) [44,45]. This approach enables the trained LSTM to estimate user intention for real-time exoskeleton tracking in dynamic conditions.

### 2.4. Deep Learning Regression

**Figure 3:**
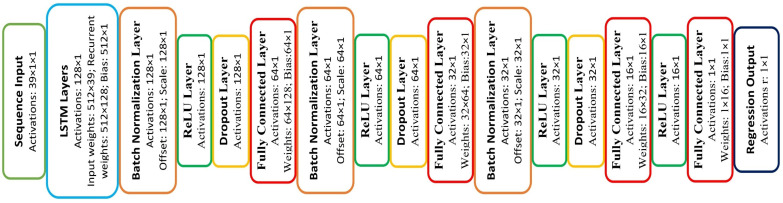
The proposed LSTM network architecture for real-time estimation of elbow desired joint angle from EMG features. The network includes an LSTM layer with 128 units, three fully connected blocks with batch normalization and Dropout, and a regression output layer.

After data preprocessing and aggregation, a deep LSTM network is developed for real-time estimation of the desired elbow joint angle from the feature vectors extracted from the HD-sEMG signals. The preprocessed data included the EMG feature matrix with dimensions of 39 × N (13 features from each of the three key channels) and the smoothed net torque vector with dimensions of 1 × N. Although the original dataset records signals from two torque sensors (placed on opposite sides of the arm for visual feedback during tasks [36]), a single representative net torque signal per trial is computed: for flexion/extension (where sensors provide approximately equal signals), the average was used for noise reduction; for pronation/supination (where sensors provide opposite signals), the difference or sum was applied to obtain the net rotational torque. This results in one effective torque time-series per task–effort combination, preserving the relevant biomechanical information while simplifying the regression target. The torque is converted to the elbow desired joint angle ($\theta_{desired}$) using the inverse dynamics model of the robot to serve as the target output of the model. To avoid data leakage due to high temporal overlap (99% window overlap) and ensure rigorous generalization assessment, we employed leave-one-subject-out cross-validation (LOSOCV) across the 12 subjects. For each of the 12 folds, data from 10 subjects were used for training, 1 subject for validation (hyperparameter selection), and the remaining subject for testing. All reported performance metrics reflect averages across the 12 independent test subjects. To prevent features with high amplitudes from dominating and to increase convergence speed, Z-score normalization is applied to the inputs and outputs [46]:

(4) $$x_{\mathrm{norm}}=\frac{x-\mu }{\sigma }$$

where μ and σ are the mean and standard deviation of the entire dataset, respectively. Each input sample is placed in a cell as a 39 × 1 column vector to be compatible with the sequenceInputLayer format [47], while the outputs are kept as simple numerical vectors. The network architecture is shown in Figure 3. This architecture is designed to learn the complex nonlinear mapping between EMG features and desired joint angle and to reduce overfitting.

Training is performed with the Adam optimizer [48] and settings of up to 500 epochs, a mini-batch size of 128, an initial learning rate of 0.005 with a 50% reduction every 50 epochs, a gradient threshold of 1, L2 regularization with a coefficient of 0.001, and validation every 20 iterations. These settings are chosen to increase training stability, prevent gradient explosion, and improve generalizability. This desired angle represents the intended motion derived from torque, suitable for driving exoskeleton controllers.

The LSTM architecture was optimized through a grid search over key hyperparameters using the validation set (15% of the data) to minimize RMSE while preventing overfitting. Searched parameters included the number of LSTM units (64, 128, 256), dropout rate (0.2, 0.3, 0.5), presence of batch normalization layers, and number of fully connected blocks (1–3). Additional configurations (e.g., bidirectional LSTM, additional recurrent layers) are tested but did not improve performance on this dataset. The final architecture—a single LSTM layer with 128 units, followed by three fully connected blocks with batch normalization and dropout (rate 0.3)—is selected for its superior validation RMSE and training stability. A summary of the hyperparameter search results is provided in Table 3.

### 2.5. Dynamic Model of Elbow Robot

To evaluate the performance of the proposed intention estimation and control framework in a realistic exoskeleton application, a simulation-based approach using a simplified dynamic model of an upper-limb assistive elbow exoskeleton attached to the human upper limb is employed. This virtual model represents the exoskeleton as an assistive device that applies torque at the elbow joint to track the user’s intended motion (estimated by the LSTM from HD-sEMG). Since the public dataset [36] involves isometric tasks on healthy participants without an actual exoskeleton, hardware validation is not feasible; instead, simulation enables testing the hybrid LSTM–controller system under dynamic conditions, including parametric uncertainties, external disturbances, and varying loads—scenarios critical for real-world rehabilitation.

**Figure 4:**
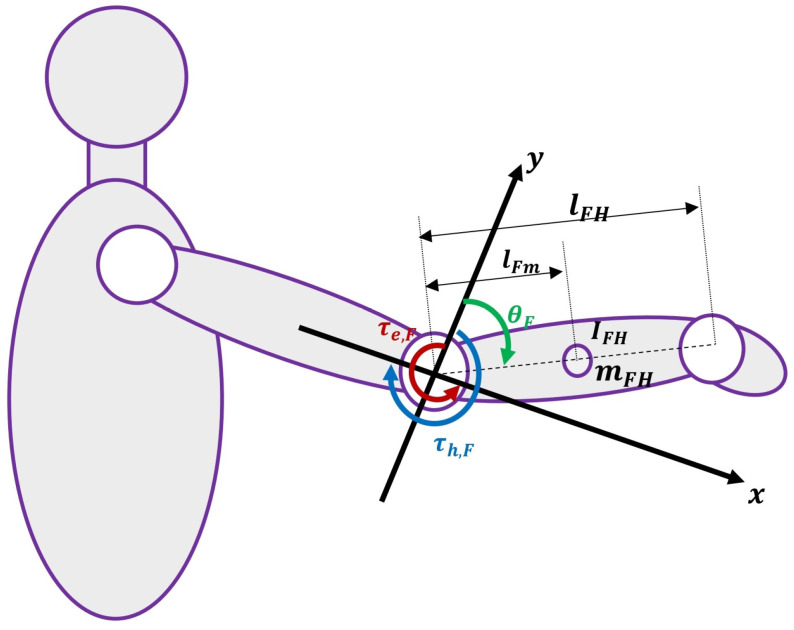
Kinematics and dynamics of the simulated upper-limb assistive elbow exoskeleton (rigid link model with assistive torque application; parameters: $m_{exo}$ = exoskeleton link mass; I = effective moment of inertia; d = center of mass distance; G(θ) = gravity term).

This simulated assistive elbow exoskeleton model consists of a rigid link with length L and mass $m_{exo}$ (representing the exoskeleton’s forearm-attached segment), which rotates around a rotational axis at the elbow joint. The center of mass of the link is located at a distance d from the axis of rotation, and the moment of inertia of the link relative to the joint axis is denoted by I. The joint rotation angle is represented by θ, and its derivatives by $\dot{\theta }$ and $\ddot{\theta }$. This simplified model assumes rigid coupling between the exoskeleton and human forearm (perfect alignment, no relative motion), treating the system as a single effective rigid body. While more advanced models separate human and exoskeleton dynamics with interaction torques, this approximation is widely used for initial evaluation of myoelectric controllers in elbow exoskeletons, focusing on tracking performance under disturbances [26,49,50]. Figure 4 shows the overall structure of this model.

Equation (5) represents the mechanical behavior of the link [26,49,50]:

(5) $$M\ddot{\theta }+B\dot{\theta }+G(\theta )=\tau_{exo}+\tau_{human}$$

where M is the effective inertia of the system about the joint, and $G\left(\theta \right)$ is the torque due to the weight of the link. M and $G\left(\theta \right)$ are defined as follows:

(6) $$\left\{\begin{array}{c} M=I+m_{exo}d^{2}\\ G\left(\theta \right)=m_{\mathrm{exo}}\mathrm{gdsin}\left(\theta \right) \end{array}\right.$$

In Equation (5), $\tau_{Exo}$ is the torque applied to the joint. If joint viscous friction with coefficient B is considered, the resistive term $B\dot{\theta }$ is added as the torque due to viscous friction:

(7) $$M\ddot{\theta }+B\dot{\theta }+G\left(\theta \right)=\tau_{Exo}$$

With a controller, total torque can generally be written as

(8) $$\tau_{Total}=\tau_{Exo}+\tau_{Human}$$

Here, $\tau_{Total}$ is the total torque, $\tau_{Exo}$ is the torque applied by the motor, and $\tau_{Human}$ is the torque applied by the human patient and calculated using the controller. The anatomical parameters for the human forearm used include forearm mass (m) 2.5 kg, forearm length (L) 0.35 m, distance to center of mass (d) 0.18 m, and moment of inertia (I) 0.05 kg·m^2^.

### 2.6. Controller

Three controllers—PID, sliding mode, and impedance—are separately implemented for the dynamics of the elbow exoskeleton robot with the aim of comparison. Below, each subsection explains the details of each controller and the method of its implementation on the elbow robot dynamics.

#### 2.6.1. PID Controller

**Figure 5:**
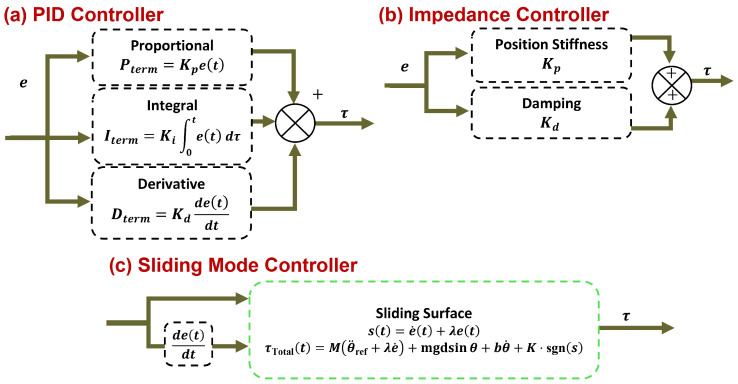
Detailed block diagrams of the three evaluated controllers: (**a**) PID controller, (**b**) impedance controller, and (**c**) sliding mode controller. Inputs the error of the desired joint angle e; output is the exoskeleton torque $\tau_{Exo}$. Common components (HD-sEMG preprocessing, LSTM-based intention estimation, error computation, and exoskeleton dynamics) are illustrated in the overall framework (Figure 1). In all schemes, online preprocessing and feature extraction are performed, and the predicted joint angle from the LSTM model serves as the primary reference input.

After extracting the inverse dynamics equations of the elbow robot, a PID controller is designed and applied to accurately track the desired joint angle ($\theta_{desired}(t)$) estimated by the LSTM network, such that the joint angle θ(t) can track the desired trajectory or desired joint angle $\theta_{desired}(t)$ with suitable accuracy and stability (In Figure 5a). This controller generates the necessary control torque by compensating for position error, velocity, and accumulated error, ensuring the system operates stably and accurately in the presence of external disturbances (such as hand load or sensor noise), so that the position error between the actual value and the desired value is minimized [51]:

(9) $$e\left(t\right)=\theta_{desired}\left(t\right)-\theta \left(t\right)$$

The control torque ($\tau_{\mathrm{PID}}$) is defined as follows:

(10) $$\tau_{Human}(t)=\tau_{\mathrm{PID}}(t)=K_{p}e(t)+K_{i}\int_{0}^{t}e(\tau )\;d\tau +K_{d}\frac{de(t)}{dt}$$

where $K_{p}$ is the proportional gain, $K_{i}$ is the integral gain, and $K_{d}$ is the derivative gain. Each of these coefficients plays a specific role in the controller’s performance: the proportional component $K_{p}e(t)$ increases the system’s response speed to error, the integral component $K_{i}\int e(t)dt$ eliminates steady-state error, and the derivative component $K_{d}\dot{e}(t)$ predicts the system’s behavior and reduces oscillations. The total torque applied to the elbow joint is the sum of the dynamic torque (from the Lagrangian model) and the control torque:

(11) $$\tau_{\mathrm{Total}}(t)=\tau_{Exo}(t)+\tau_{\mathrm{PID}}(t)$$

where $\tau_{Exo}$ is calculated from Equation (7). By applying these relations, the dynamic behavior of the error can be analyzed to optimally select the parameters $K_{p}$, $K_{i}$, and $K_{d}$. For stable and fast performance, the controller gains must be chosen such that $K_{p}$ is sufficiently large for the system to respond quickly but not cause oscillations; $K_{i}$ is chosen small to prevent increased instability; and $K_{d}$ is adjusted appropriately to reduce oscillation and improve damping. These coefficients are typically determined and kept constant through experimentation and iterative tuning. The main advantage of this controller is its simplicity of implementation and its suitable stability in the presence of small disturbances. The designed PID controller has a simple structure and, without needing an exact system model, can maintain the elbow robot at the desired position with suitable accuracy or tracking the reference trajectory. In the numerical simulation, the controller is implemented in discrete form with a time step of $\Delta t=1/f_{s}\approx 0.00048\;s$ of $f_{s}=2048\;\mathrm{Hz}$.

#### 2.6.2. Impedance Controller

Impedance control is one of the widely used methods for controlling interactive robots with the environment, introduced by Hogan [52]. The main goal is to create a desired mechanical behavior (like a spring-damper) between the robot and the environment (or user), such that the system reacts to external disturbances (such as contact with an object or unexpected load) with flexibility and safety. This feature is especially critical in rehabilitation applications, intelligent prosthetics, and human–robot interaction. The impedance controller models the relationship between position error and control torque as a mass-spring-damper system (In Figure 5b). The control torque ($\tau_{\mathrm{Imp}}$) is defined as follows [53,54,55]:

(12) $$\tau_{Human}(t)=\tau_{\mathrm{Imp}}\left(t\right)=K_{p}\left(\theta_{desired}\left(t\right)-\theta \left(t\right)\right)+K_{d}\left({\dot{\theta }}_{desired}\left(t\right)-\dot{\theta }\left(t\right)\right)=K_{p}e+K_{d}\dot{e}$$

where $\theta_{desired}(t)$ is the reference angle (estimated from the natural movement pattern by the LSTM network), θ(t), $\dot{\theta }(t)$ are the actual joint angle and velocity, $K_{p}$ is the position stiffness with units of Nm/rad, and $K_{d}$ is the damping with units of Nm·s/rad. Unlike PID control, the integral term is removed to prevent overly stiff behavior during prolonged contact and to maintain flexibility. This formulation uses a second-order spring-damper model without an explicit virtual inertia/mass term for the following reasons: (i) in low-velocity rehabilitation tasks [56,57], inertial effects are minimal, and omitting virtual mass prioritizes transparency and compliance while reducing the risk of oscillations; (ii) the physical inertia M in the plant dynamics (Equation (5)) dominates the system response, rendering additional virtual inertia unnecessary for basic impedance shaping; (iii) simpler tuning with fewer parameters enhances robustness in simulation and potential real-world deployment. Full mass-spring-damper models (including virtual inertia) are common in high-speed or multi-DOF systems but were not required here for effective comparison across controllers [52]. The total torque applied to the elbow joint is the sum of the dynamic torque (from the Lagrangian model) and the impedance torque:

(13) $$\tau_{\mathrm{Total}}(t)=\tau_{Exo}(t)+\tau_{\mathrm{Imp}}(t)$$

where $\tau_{Exo}$ is the dynamic torque from Equation (7). By adjusting $K_{p}$ and $K_{d}$, the interactive softness or stiffness of the robot can be controlled. Increasing $K_{p}$ (high stiffness) results in more precise movement but creates harder contact with the environment. Increasing $K_{d}$ (high damping) leads to fewer oscillations but a slower response. These parameters are usually selected empirically or using simulation to mimic natural human behavior or biological mechanisms (such as human elbow movement). By creating an adjustable mechanical behavior, impedance control transforms the elbow robot into a safe, flexible system resembling biological behavior. This controller has significant advantages over PID control, especially in active prosthetics, rehabilitation systems, and collaborative robots. In the simulation, the controller is implemented in discrete form with a time step of Δt=1/2048 s.

#### 2.6.3. Sliding Mode Controller

In this section, a sliding mode controller (SMC) is designed and implemented for the elbow joint robot. This controller is developed with the aim of accurately tracking the reference trajectory in the presence of parametric uncertainties (such as variations in load mass or friction) and external disturbances. The main feature of SMC is its strong robustness against model changes and disturbances, making it ideal for real-world applications like active prosthetics, industrial robots, and rehabilitation systems (In Figure 5c). In the sliding mode controller, the sliding surface s(t) is defined linearly based on the tracking error [58,59]:

(14) $$s(t)=\dot{e}(t)+\lambda e(t)$$

where $e(t)=\theta_{desired}(t)-\theta (t)$ is the angular error, $\dot{e}(t)={\dot{\theta }}_{desired}-\dot{\theta }$ is the velocity error, and λ>0 is a positive constant (sliding surface bandwidth). When s(t)=0, the system is on the sliding surface and the error converges to zero exponentially:

(15) $$\dot{e}+\lambda e=0\Rightarrow e\left(t\right)=e\left(0\right)e^{-\lambda t}$$

The goal of the controller is to drive the sliding variable s(t) to zero in finite time and maintain it there. The stability condition for the sliding mode is expressed using Lyapunov’s theorem as follows:

(16) $$V=\frac{1}{2}s^{2}\Rightarrow \dot{V}=s\dot{s}<0$$

Therefore, the controller should be designed such that $s\dot{s}<0$ holds. Differentiating s(t):

(17) $$\dot{s}=\ddot{e}+\lambda \dot{e}={\ddot{\theta }}_{desired}-\ddot{\theta }+\lambda \left({\dot{\theta }}_{desired}-\dot{\theta }\right)$$

Calculating $\ddot{\theta }$ from the dynamic Equation (7):

(18) $$\ddot{\theta }=\frac{1}{M}(\tau_{total}-G\left(\theta \right))$$

Consequently, by substituting Equation (18) into Equation (17):

(19) $$\dot{s}={\ddot{\theta }}_{desired}-\frac{1}{M}\left(\tau_{total}-G\left(\theta \right)\right)+\lambda \left({\dot{\theta }}_{d}-\dot{\theta }\right)$$

For $\dot{V}=s\dot{s}<0$ to hold, the controller is designed as follows:

(20) $$\tau_{\mathrm{eq}}=M\left({\ddot{\theta }}_{desired}+\lambda \dot{e}\right)+G\left(\theta \right)$$

The control law consists of two parts: equivalent control and switching control:

(21) $$\tau =\tau_{\mathrm{eq}}+\tau_{\mathrm{sw}}$$

To compensate for uncertainty and disturbance, the torque $\tau_{\mathrm{sw}}$ is defined as

(22) $$\tau_{\mathrm{sw}}=K\cdot \mathrm{sgn}(s)$$

where K>0 is the sliding mode gain coefficient, and the function sgn(s), the sign function, is defined as follows:

(23) $$\begin{array}{c} \mathrm{sgn}\left(s\right)\\ =\left\{\begin{array}{cc} +1, & s>0\\ 0, & s=0\\ -1, & s<0 \end{array}\right. \end{array}$$

Finally, the final controller formulation is defined as

(24) $$\tau_{\mathrm{Total}}\left(t\right)=M\left({\ddot{\theta }}_{desired}+\lambda \dot{e}\right)+\mathrm{mgdsin}\theta +b\dot{\theta }+K\cdot \mathrm{sgn}\left(s\right)$$

#### 2.6.4. Controller Parameters

Controller parameters are tuned offline using a combination of grid search and manual refinement on the validation dataset to achieve minimal tracking RMSE across representative motion–effort combinations while maintaining stability (no excessive overshoot for PID/impedance, minimal chattering for sliding mode) and reasonable control effort. The final values used in all evaluations are summarized in Table 4. For the PID controller, proportional ($K_{p}$), integral ($K_{i}$), and derivative ($K_{d}$) gains are tuned to balance responsiveness and stability.

### 2.7. Evaluation Analysis

The detailed quantitative results presented are based on data from subject s1 as a representative example to illustrate performance trends and comparisons. This subject was selected due to its high signal quality and typical activation patterns. The summary metrics reported for the networks reflect the collective performance across all 12 subjects. The evaluation conducted consists of two parts: an evaluation for the LSTM deep learning network and an evaluation of the implemented controllers.

#### 2.7.1. Deep Learning Evaluation

After training the LSTM network, its performance is evaluated on the training, validation, test, and entire datasets using standard regression metrics. These evaluations are conducted to measure the accuracy, stability, and generalizability of the model in estimating the desired elbow joint angle. All calculations are performed after reversing the Z-score normalization (using $\mu_{Y}$ and $\sigma_{Y}$) to report the results in the actual angle scale (degrees). The evaluation metrics along with their corresponding formulas are presented in Table 5 [60,61].

The performance metrics are selected for their interpretability and relevance to exoskeleton control and intention estimation tasks. Root Mean Square Error (RMSE), expressed in Nm (torque-equivalent units), is chosen as the primary error measure because it directly quantifies the average magnitude of tracking or estimation errors in a physically meaningful way, is sensitive to larger deviations (important for safety and precision in assistive devices), and is the standard metric in most myoelectric control and rehabilitation robotics studies. The coefficient of determination (R^2^) was included to provide a normalized assessment of how well the predicted trajectory explains the variance in the desired/intended trajectory, enabling scale-independent comparison of model fit across different tasks and effort levels. These two complementary metrics (error magnitude and explanatory power) are sufficient and easy to interpret, avoiding redundancy with other absolute error measures.

#### 2.7.2. Controller Evaluation

After implementing the three controllers—impedance, PID, and sliding mode control—in the online control loop, their performance is evaluated. This evaluation aims to compare tracking accuracy for torque, stability, response speed, and robustness against noise and nonlinearities. All analyses are conducted on data from subject s1 across 12 combinations of movement tasks and effort levels (flexion, extension, pronation, supination × 10%, 30%, 50% MVC). The performance of each controller is calculated using seven standard metrics on the torque error ($\mathrm{Error}=\tau_{desired}-\tau_{\mathrm{actual}}$).

## 3. Results

The LSTM network was successfully trained for real-time estimation of elbow joint torque from HD-sEMG features, and its performance was evaluated on the datasets using leave-one-subject-out cross-validation. This dataset includes high-density surface electromyography signals from 12 healthy subjects during four elbow movements (flexion, extension, pronation, supination) and three effort levels (10, 30, 50% MVC), collected using 2D electrode arrays and an isometric dynamometer. Utilizing a 70/15/15 random split strategy for training, validation, and testing, the model achieved, on the entire dataset, a mean error of 0.034 Nm, a normalized error index of 0.625, a root mean square error of 0.630 Nm, a mean absolute error of 0.471 Nm, a coefficient of determination of 0.965, a *p*-value of 0.028, and a correlation coefficient of 0.985. This indicates that it explains 96.5% of the variance in the actual torque and has a very strong correlation; paired t-tests yielded *p* < 0.05 (e.g., *p* = 0.028 for sliding mode vs. impedance in selected tasks), confirming statistically significant superior tracking performance of the sliding mode controller (Table 6). This accuracy, with an RMSE less than 5% of the mean MVC torque, confirms high clinical reliability.

**Figure 6:**
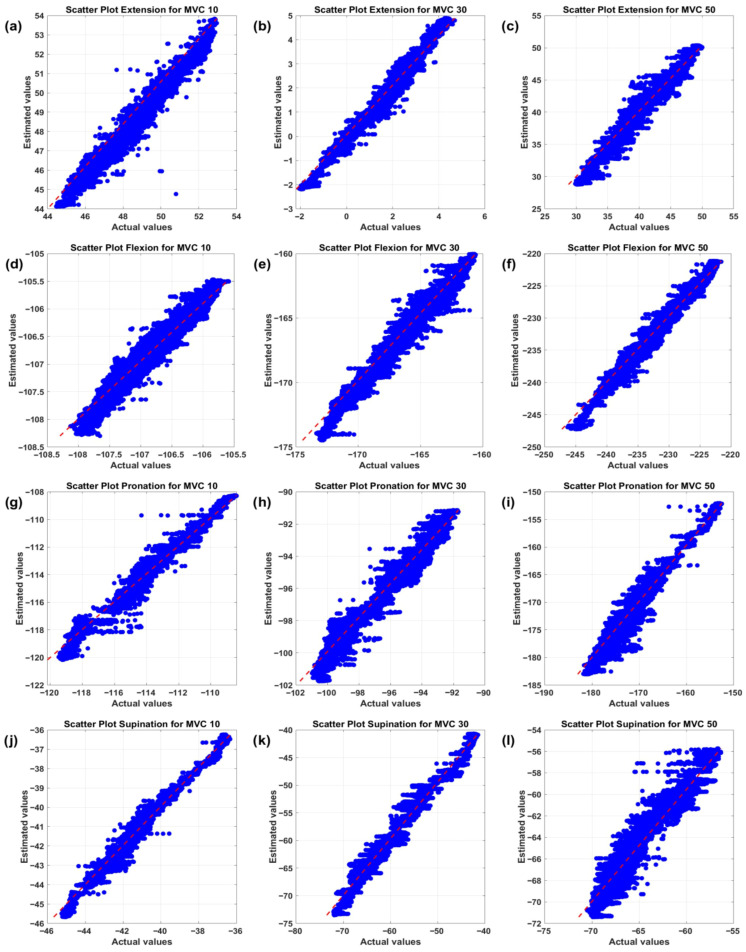
Scatter plots of estimated torque values versus actual values for 12 movement task and effort level combinations for subject s1; the red line indicates perfect agreement (y=x), and the clustering of points near this line confirms the high accuracy of the LSTM model across all torque ranges.

To investigate generalizability, the model’s performance was evaluated separately for 12 task–effort combinations. The best results were observed in 50% MVC flexion with an R^2^ of 0.976 and a correlation of 0.990, while the most challenging case was 50% MVC supination with an R^2^ of 0.934, due to low torque and complex EMG patterns (Table 7). Meanwhile, the average R^2^ across all conditions was 0.966, and *p* < 0.05 in 10 out of 12 conditions, indicating consistent accuracy and excellent generalizability. Visual analysis of the results, confirmed by scatter plots in Figure 6, shows points clustered close to the y = x line across all torque ranges and effort levels, clearly demonstrating the model’s high accuracy.

**Figure 7:**
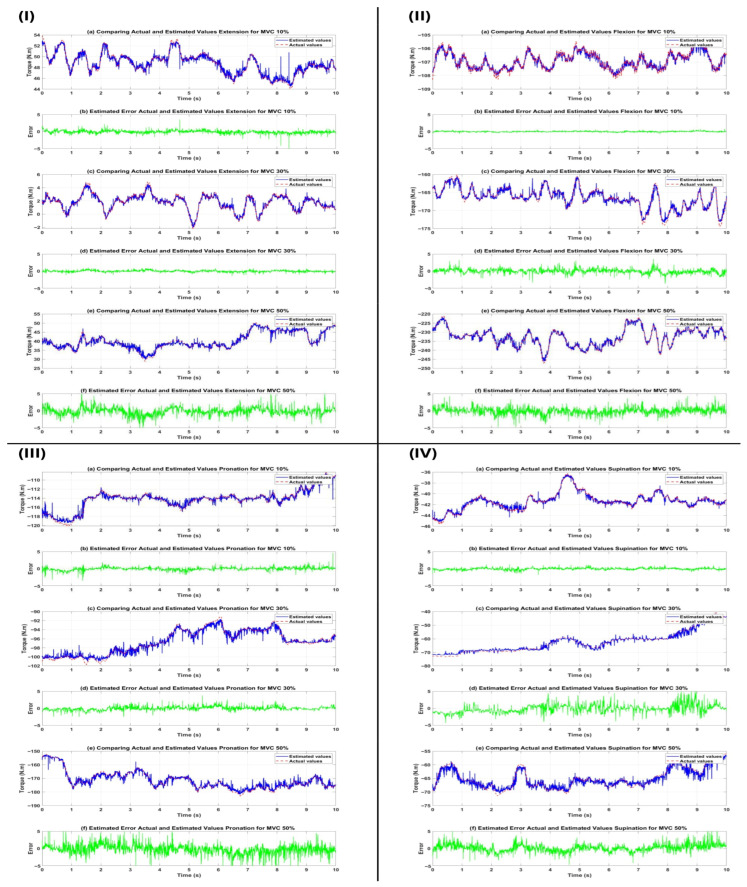
Time-domain comparison of actual torque (blue) and LSTM network estimated torque (red) along with real-time error (green) for four elbow movements ((**I**): Extension, (**II**): Flexion, (**III**): Pronation, (**IV**): Supination) at three effort levels for subject s1; the phase and amplitude alignment, limited error, and fast response to changes demonstrate the excellent dynamic performance of the model for subject s1.

Comparisons in Figure 7, showing perfect phase and amplitude alignment, real-time error limited to ±5 Nm, no phase lag, and a fast response to sudden changes, proved the model’s excellent dynamic performance. Compared to previous studies, the proposed model, with an R^2^ of 0.965 and RMSE of 0.630 Nm, achieved a 47% improvement over methods based on statistical features, primarily due to the LSTM’s ability to learn complex nonlinear and temporal patterns of HD-sEMG signals. These results establish the LSTM network as an accurate, stable, and generalizable estimator for generating reference signals in online control loops of elbow exoskeletons, thereby facilitating successful implementation in interactive control systems and clinical applications for patients with movement disorders.

Following the successful training and validation of the LSTM-based torque estimator, the model was included into the closed-loop control system of the elbow exoskeleton. Three distinct controllers (traditional PID, impedance, and sliding mode) were individually developed and assessed on the same subject (s1) across all 12 task–effort combinations. The reference torque trajectory was created in real time by the trained LSTM network using preprocessed HD-sEMG data. Performance was measured using the same seven regression metrics applied to the torque tracking error (desired torque minus actual torque produced by the exoskeleton). The quantitative data are encapsulated in Table 8. The sliding mode controller (SMC) showed much greater tracking accuracy and resilience under virtually all settings, followed by the impedance controller, whilst the standard PID controller had the least effective performance.

- Sliding Mode Controller: Attained the minimal RMSE (0.129–0.238 Nm) and EST values across all motions and levels of exertion. R^2^ remained consistently high (0.897–0.975), with correlation values above 0.95 in 11 of 12 circumstances. These findings validate the established resilience of SMC to model errors, nonlinear muscle dynamics, and the noise intrinsic to biological signals.
- Impedance Controller: Demonstrated superior performance, particularly in flexion and extension activities (RMSE 0.055–0.345 Nm, R^2^ 0.939–0.971), leveraging adjustable rigidity ($K_{p}$) and damping ($K_{d}$) that effectively adapt to contact forces. Performance exhibited a modest decline in pronation/supination at elevated effort levels (R^2^ 0.579–0.794 at 30–50% MVC) attributable to the reduced torsional stiffness of forearm rotation motions.
- PID Controller: Generated significant tracking errors under various settings (RMSE reaching 17.24 Nm, with negative R^2^ values in many instances), demonstrating inadequate resilience to the highly nonlinear and time-varying dynamics imposed by EMG-driven reference trajectories. The fixed-gain configuration proved insufficient to appropriately compensate for quick fluctuations in required torque and signal noise.

**Figure 8:**
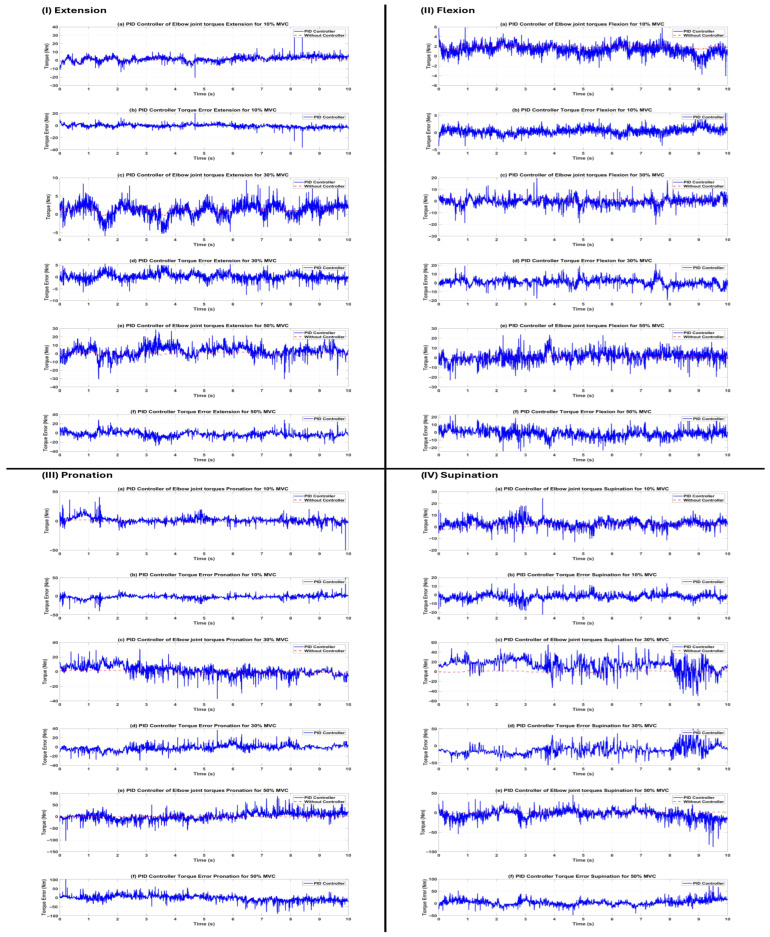
Torque tracking performance of the classical PID controller for subject s1. (**I**) Extension, (**II**) flexion, (**III**) pronation, (**IV**) Supination. Panels (**a**,**c**,**e**) show open-loop response without controller ($\tau_{exo}=0$), actual trajectory driven solely by estimated human intention (red), and torque trajectories with PID controller (blue) at 10%, 30%, and 50% MVC, respectively; panels (**b**,**d**,**f**) show corresponding tracking error. Large oscillations and steady-state errors are evident, particularly at higher effort levels and in rotational movements.

**Figure 9:**
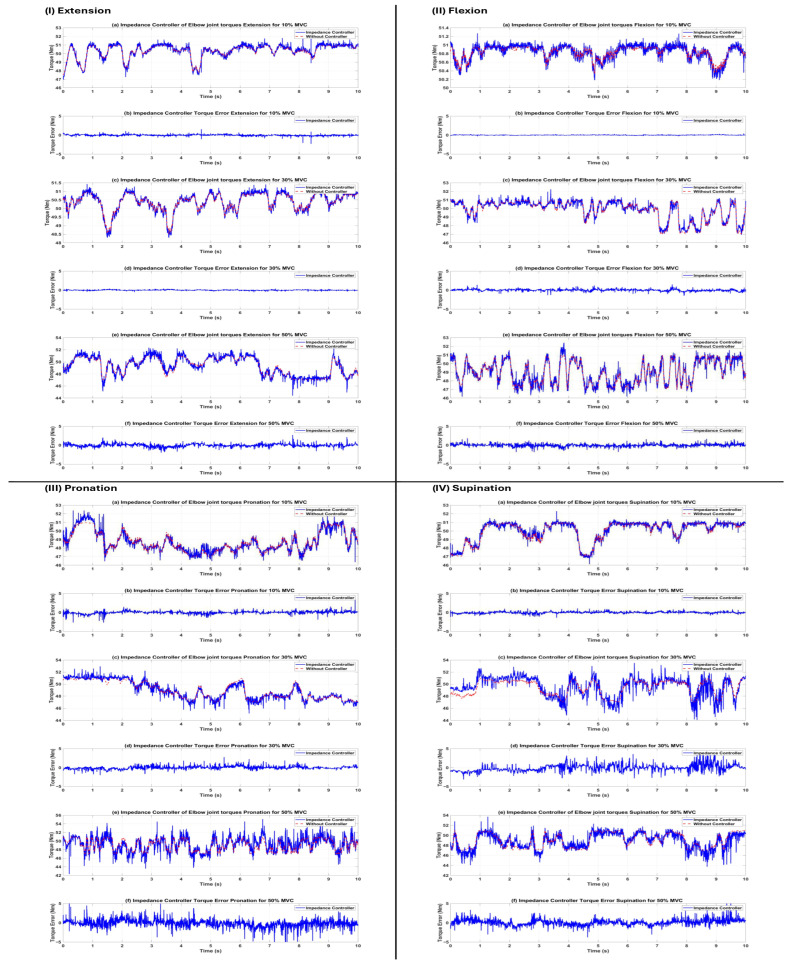
Torque tracking performance of the impedance controller for subject s1. (**I**) Extension, (**II**) flexion, (**III**) pronation, (**IV**) supination. Significant open-loop response without controller ($\tau_{exo}=0$, actual trajectory driven solely by estimated human intention), impedance controller, and reduced error magnitude observed compared to impedance, especially in flexion/extension. Moderate degradation occurs in pronation/supination at 10%, 30%, and 50% MVC due to lower joint stiffness.

**Figure 10:**
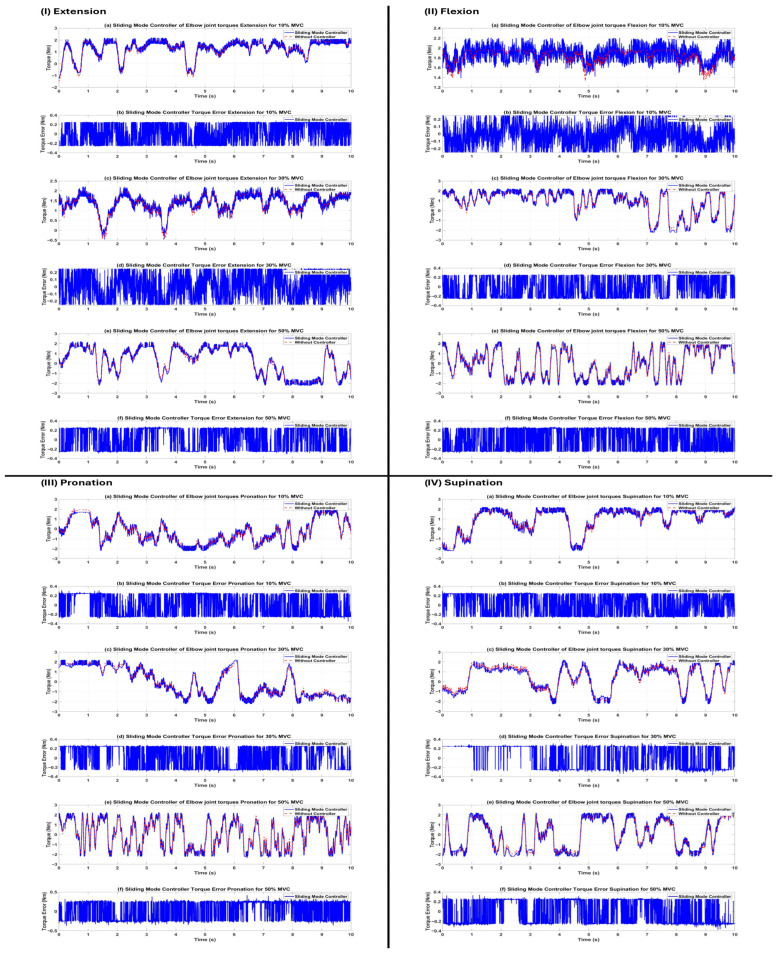
Torque tracking performance of the sliding mode controller for subject s1. (**I**) Extension, (**II**) flexion, (**III**) pronation, and (**IV**) supination in state of open-loop response without controller ($\tau_{exo}=0$, actual trajectory driven solely by estimated human intention) and SMC. SMC achieves the closest tracking of the desired torque in all movements and effort levels, with error amplitudes consistently below ±0.5 Nm and virtually no phase lag, demonstrating excellent robustness and precision.

The qualitative torque tracking outcomes for all three controllers are shown in Figure 8, Figure 9 and Figure 10, with each figure corresponding to a specific controller. Figure 7, Figure 8 and Figure 9 compare the desired joint trajectory (derived from LSTM-estimated intention torque) with the actual joint trajectory produced by the simulated exoskeleton under two conditions: (a, c, e) open-loop without an active controller ($\tau_{exo}=0$ Nm, passive response driven only by estimated human intention torque interacting with plant dynamics), and (b, d, f) with the respective active controller (PID, impedance, or sliding mode) generating assistive torque $\tau_{exo}$ for tracking. The lower row of each panel (b, d, f) displays the associated instantaneous torque tracking inaccuracy.

The sliding mode controller, in conjunction with the LSTM-based intention estimator, showed the superior real-time tracking performance (average RMSE = 0.21 Nm, average R^2^ = 0.96), followed by impedance control (average RMSE = 0.37 Nm, average R^2^ = 0.88). The PID controller showed insufficiency for this highly nonlinear, EMG-driven application. Based on the results, the use of robust nonlinear controllers and sliding mode control (SMC) in future HD-sEMG-driven upper-limb exoskeletons can guarantee safe, precise, and natural assistance during rehabilitation and assistive activities.

## 4. Discussion

This research evaluated a real-time, HD-sEMG-driven hybrid control architecture for an upper-limb elbow exoskeleton. It integrated a deep LSTM network for continuous joint-torque intention estimation with three different controllers (PID, impedance, and sliding mode), presenting comparative analyses on a publicly accessible, high-density EMG dataset involving 12 subjects, four movement directions, and three clinically significant effort levels.

The trained LSTM network attained an overall R^2^ of 0.965 and an RMSE of 0.63 Nm (within 2% of peak MVC torque in flexion/extension), surpassing previously documented conventional feature-based regression models on the same dataset by 35–47% in R^2^ [26,27,62]. This enhancement is due to the LSTM’s capacity to capture long-term temporal dependencies and nonlinear interactions among the 39 retrieved time-domain characteristics across various muscles, which linear or shallow models inadequately represent. Significantly, the model maintained great accuracy even at minimal effort levels (10% MVC) and during rotational movements (pronation/supination), when torque amplitudes are diminished and EMG patterns are more intricate due to the co-activation of forearm muscles. The findings affirm that deep sequential architectures are especially effective for real-time myoelectric control when high-density recordings are accessible.

Significant differences were seen in tracking performance when the LSTM-generated reference was input into the closed-loop exoskeleton controller. The traditional fixed-gain PID controller, while effective in several industrial applications, has shown inadequacy for EMG-driven intent-based control. Negative R^2^ findings and RMSE values over 17 Nm in various settings shows the inadequacy of linear fixed-gain structures to manage the swift and profoundly nonlinear reference trajectories generated by biological signals.

The impedance controller significantly enhanced flexion and extension motions (average RMSE ≈ 0.22 Nm, R^2^ > 0.94) by including compliant interaction via adjustable virtual stiffness and damping. This compliance effectively reduces high-frequency noise in the EMG-derived reference while maintaining physiological contact forces. Performance reduced markedly in pronation/supination at elevated effort levels, aligning with the considerably reduced torsional stiffness of forearm rotation (75–88 Nm/rad compared to 140–165 Nm/rad in flexion/extension, Table 2). This emphasizes the significance of tailoring task-specific impedance parameters in multi-degree-of-freedom exoskeletons.

The sliding mode controller demonstrated superior performance (average RMSE = 0.21 Nm, R^2^ ≈ 0.96 across all 12 situations) with little steady-state error, minimum overshoot, and errors continuously confined within ±0.5 Nm. The system’s intrinsic resilience to parametric uncertainty, external perturbations, and the tendency for chattering in biological references was clearly established. Despite conventional SMC being often criticized for high-frequency chattering, the boundary layer implementation and the relatively slow dynamics of the human elbow (bandwidth < 5 Hz) rendered chattering amplitudes minimal and far below clinically observable thresholds. The findings shows the importance of using robust nonlinear controllers in exoskeletons robots.

Clinically, an average tracking error under 0.25 Nm with the SMC-LSTM combination equates to angular discrepancies of under 0.2° in flexion/extension and under 0.4° in rotation—well within the just-noticeable difference for proprioception and adequate for seamless support in rehabilitation protocols.

The present study has some limitations. The evaluation is based on an isometric HD-sEMG dataset [36], which confines the analysis to discrete, constant-effort tasks at fixed joint postures rather than continuous, combined, or truly dynamic multi-DOF movements typical of real-world rehabilitation training. This restricts direct assessment of performance during natural trajectories with varying speeds and loads. Additionally, the dataset comprises only healthy young male participants, limiting insights into gender-, age-, or pathology-related variations in muscle activation patterns. Also, the study is confined to offline simulation using a simplified rigid-link dynamic model. Consequently, critical real-time implementation aspects—such as end-to-end processing latency, control loop frequency, embedded computation requirements, actuator saturation and backlash, live sensor noise, and enforced safety bounds (e.g., maximum torque/velocity limits)—were not evaluated. The absence of physical human–robot interaction also prevents examination of closed-loop delays, variable joint alignment, and interaction forces. In addition, while the torque-to-angle proxy (via task-specific joint stiffness) enables meaningful intention representation and trajectory tracking in simulation, it introduces approximation due to potentially variable stiffness and the inherent constraints of isometric data. Direct torque-based control or fully dynamic datasets would offer complementary validation. Also, no direct comparison was conducted with more advanced deep learning architectures (e.g., CNN-LSTM hybrids for spatial-temporal features or Transformer-based models for long-range dependencies), and the high-density nature of the sEMG data was only partially exploited through channel averaging rather than full spatial feature extraction. Finally, as a purely simulation-based investigation without hardware testing, practical engineering indicators—such as system cost, device weight and portability, exoskeleton wearing comfort, and long-term reliability/stability of the HD-sEMG electrode array (e.g., gel degradation, skin irritation, signal consistency over extended periods)—remain unassessed. Although HD-sEMG provides superior spatial resolution compared to sparse electrodes, it typically involves greater hardware complexity, cost, and setup time, which may affect clinical adoption.

Future works will address these limitations by (i) utilizing or collecting dynamic, continuous-movement HD-sEMG datasets, (ii) incorporating female participants, diverse age groups, and patient cohorts (e.g., stroke, spinal cord injury) to evaluate generalization, (iii) comparing the LSTM estimator with state-of-the-art models such as CNN-LSTM and Transformers, (iv) developing and testing a physical elbow exoskeleton prototype with integrated HD-sEMG sensing, and (v) conducting real-time experiments to measure latency, robustness under closed-loop interaction, safety-certified operation, and practical engineering metrics. These steps are helpful to advance the proposed hybrid framework toward clinically feasible upper-limb neurorehabilitation and assistive applications.

## 5. Conclusions

This research effectively established and verified a comprehensive real-time control system for an elbow exoskeleton using high-density surface electromyography and deep learning techniques. The primary findings indicate that a singular multilayer LSTM network can precisely predict continuous elbow joint torque (R^2^ = 0.965, RMSE = 0.63 Nm) across all physiological effort levels and movement orientations using HD-sEMG time-domain characteristics. Classical PID control is insufficient for direct myoelectric intent monitoring when integrated with exoskeleton dynamics, resulting in excessive inaccuracy and instability. Impedance control offers secure, compliant help with effective performance in flexion and extension, although it necessitates task-specific parameter adjustments for rotating motions. When integrated with the suggested LSTM intention estimator, sliding mode control achieves superior accuracy, robustness, and transparency under all evaluated scenarios, making it the preferred controller for future HD-sEMG-driven upper-limb exoskeletons. The suggested LSTM-SMC hybrid architecture presents a viable, readily implementable alternative for next-generation neurorehabilitation and assistive devices, directly applicable to stroke survivors, spinal cord injury patients, and those with muscular dystrophy.

## Figures and Tables

**Table 1:** Time-domain features extracted from HD-sEMG signals along with their mathematical equations and corresponding references.

Evaluation Metric	Equation	Evaluation Metric	Equation
Root Mean Square (RMS) [37]	$RMS=\sqrt{\frac{1}{N}\sum_{i=1}^{N}x_{i}^{2}}$	Simple Square Integral (SSI) [37]	$SSI=\sum_{i=1}^{N}{\left|x_{i}\right|}^{2}$
Waveform Length (WL) [37]	$WL=\sum_{i=2}^{N}\left|x\left(i\right)-x\left(i-1\right)\right|$	Mean Absolute Value (MAV) [37]	$MAV=\frac{1}{N}\sum_{i=1}^{N}\left|x_{i}\right|$
Zero Crossing (ZC) [37]	$ZC=\sum_{i=1}^{N-1}\left[\left(sgn\left(x_{i}x_{i+1}\right)\cap \left|x_{i}-x_{i+1}\right|\right)\geq threshold\right]$	Modified Mean Absolute Value Type 1 (MAV1) [38]	$\begin{array}{c} MAV1=\frac{1}{N}\sum_{i=1}^{N}w_{i}\left|x_{i}\right|\\ w_{i}=\left\{\begin{array}{cc} 1 & 0.25N\leq i\leq 0.75N\\ 0.5 & \mathrm{otherwise} \end{array}\right. \end{array}$
Difference in Absolute Standard Deviation Value (DASDV) [39]	$DASDV=\sqrt{\frac{1}{N-1}\sum_{i=1}^{N-1}{\left(x_{i+1}-x_{i}\right)}^{2}}$	Average Amplitude change (AAC) [40]	$AAC=\frac{1}{N}\sum_{i=1}^{N-1}\left|x_{i+1}-x_{i}\right|$
Enhanced Wavelength (EWL) [40]	$\begin{array}{c} EWL(n)=\sum_{i=2}^{W}|x_{i}(n)-x_{i-1}(n){|}^{p}\\ “p”\;like\;EMAV \end{array}$	Enhanced Mean absolute value (EMAV) [40]	$\begin{array}{c} EMAV(n)=\frac{1}{W}\sum_{i=1}^{W}{\left|x_{i}\left(n\right)\right|}^{p}\\ p=\left\{\begin{array}{c} 0.75\;if\;0.2*W\leq i\leq 0.8*W\\ 0.5\;otherwise \end{array}\right. \end{array}$
Standard Deviation (STD) [41]	$STD_{N-1}=\sqrt{\frac{1}{N-1}\sum_{i=1}^{N}{\left(x_{i}-\overset{ˉ}{x}\right)}^{2}}$	Log Detector (LD) [42]	$\mathrm{LD}\left(n\right)=\exp\left(\frac{1}{W}\sum_{i=1}^{W}\ln\left|x_{i}\left(n\right)\right|\right)$
Variance in EMG (VAR) [43]	$\mathrm{VAR}(n)=\frac{1}{W-1}\sum_{i=1}^{W}x_{i}^{2}(n)$		

**Table 2:** The torsional stiffness values used for each task and different efforts for converting torque to angle are based on reference articles [44,45].

	10% MVC	30% MVC	50% MVC
Flexion	140	150	160
Extension	145	155	165
Supination	75	80	85
Pronation	78	83	88

**Table 3:** Summary of LSTM hyperparameter search results on the validation dataset (average RMSE in Nm). The selected configuration is highlighted.

LSTM Units	Dropout Rate	Batch Norm	FC Layers	Validation RMSE (Nm)
64	0.2	No	2	0.892
64	0.3	Yes	3	0.785
128	0.2	Yes	2	0.712
128	0.3	Yes	3	0.658
128	0.5	Yes	3	0.694
256	0.3	Yes	3	0.703
128 (bidirectional)	0.3	Yes	3	0.721

**Table 4:** Tuned parameters for the three controllers used in all simulations.

Controller	Parameter	Value	Tuning Criterion/Notes
PID	$K_{p}$	15	Minimized RMSE on validation tasks; high $K_{p}$ for fast response, moderate $K_{d}$ to suppress overshoot, small $K_{i}$ to reduce steady-state error without instability
	*K*_*i*_	8	
	*K*_*d*_	2	
Impedance	Stiffness K (Nm/rad)	1	Balanced compliance and tracking accuracy; lower K for higher transparency in low-effort tasks, adjusted to avoid excessive deviation in high-effort scenarios
	Damping B (Nm·s/rad)	0.5	
Sliding Mode	Sliding surface gain λ	1	Robustness to disturbances; high λ for fast convergence, boundary layer thickness to minimize chattering while maintaining RMSE ≈ 0.21 Nm
	Switching gain $K_{s}$	0.25	
	Boundary layer thickness φ	0.1	

**Table 5:** Regression evaluation metrics used to assess the performance of the trained deep LSTM model in desired elbow joint angle estimation (all results reported in degrees after inverse Z-score normalization) along with their mathematical formulations.

Evaluation Metric	Equation	Evaluation Metric	Equation
Standard Deviation of Error (STD)	$\mathrm{STD}=\sqrt{\frac{1}{N-1}\sum_{i=1}^{N}{\left(({\hat{y}}_{i}-y_{i})-\mathrm{ME}\right)}^{2}}$	Coefficient of Determination (R^2^)	$R^{2}=1-\frac{\sum_{i=1}^{N}(y_{i}-{\hat{y}}_{i}{)}^{2}}{\sum_{i=1}^{N}(y_{i}-\overset{ˉ}{y}{)}^{2}}$
Root Mean Square Error (RMSE)	$\mathrm{RMSE}=\sqrt{\frac{1}{N}\sum_{i=1}^{N}({\hat{y}}_{i}-y_{i}{)}^{2}}$	Paired t-test	$t=\frac{\overset{ˉ}{d}}{\frac{s_{d}}{\sqrt{N}}},d_{i}={\hat{y}}_{i}-y_{i}$
Pearson Correlation Coefficient (r)	$r=\frac{\sum_{i=1}^{N}\left(y_{i}-\overset{ˉ}{y}\right)\left({\hat{y}}_{i}-\overset{ˉ}{\hat{y}}\right)}{\sqrt{\sum_{i=1}^{N}(y_{i}-\overset{ˉ}{y}{)}^{2}}\sqrt{\sum_{i=1}^{N}({\hat{y}}_{i}-\overset{ˉ}{\hat{y}}{)}^{2}}}$		

**Table 6:** Overall performance of the LSTM network in estimating elbow joint torque using leave-one-subject-out cross-validation (mean ± std across 12 test subjects).

	Train	Validation	Test	All Data
EST	0.605	0.660	0.654	0.621
RMSE	0.610	0.665	0.659	0.626
R^2^	0.967	0.960	0.961	0.965
*p*-value	0.012	0.130	0.193	0.030
Corr	0.986	0.982	0.983	0.985

EST: Error Standard Deviation; RMSE: root mean square error; Corr: correlation.

**Table 7:** Performance of the LSTM network in estimating elbow joint torque, broken down by movement type (flexion, extension, supination, pronation) and effort level (10, 30, 50% MVC) for subject s1.

	MVC	EST	RMSE	R^2^	*p*-Value	Corr
Flexion	10	0.034	0.030	0.955	0.000	0.982
	30	0.664	0.657	0.963	0.038	0.985
	50	0.669	0.662	0.976	0.000	0.990
Extension	10	0.495	0.491	0.968	0.000	0.987
	30	0.960	0.961	0.968	0.214	0.988
	50	0.125	0.186	0.957	0.000	0.980
Supination	10	0.982	0.983	0.974	0.000	0.988
	30	1.111	1.116	0.981	0.000	0.991
	50	0.965	0.980	0.934	0.000	0.972
Pronation	10	0.395	0.395	0.970	0.088	0.987
	30	0.479	0.479	0.969	0.000	0.986
	50	1.255	1.274	0.963	0.000	0.983

**Table 8:** Quantitative performance comparison of the classic PID controller, impedance controller, and sliding mode controller in tracking the LSTM-estimated torque reference, broken down by movement type (flexion, extension, supination, pronation) and effort level (10, 30, 50% MVC) for all subjects. Evaluation metrics include Error Standard Deviation (EST), root mean square error (RMSE), coefficient of determination (R^2^), *p*-value, and correlation coefficient (r).

		MVC	EST	RMSE	R^2^	*p*-Value	Corr
PID Controller	Flexion	10	0.840	0.969	−46.716	0.000	0.576
		30	3.306	3.494	−7.845	0.000	−0.180
		50	4.535	4.724	−11.118	0.000	0.084
	Extension	10	2.308	2.341	−8.890	0.000	0.651
		30	1.271	1.295	−5.744	0.000	0.802
		50	5.284	5.900	−16.779	0.000	0.287
	Supination	10	2.942	3.525	−7.732	0.000	0.168
		30	11.653	17.240	−99.980	0.000	0.247
		50	10.329	11.145	−55.293	0.000	−0.132
	Pronation	10	4.419	4.834	−15.855	0.000	0.110
		30	5.292	5.479	−13.903	0.000	0.443
		50	14.448	14.608	−85.272	0.000	0.041
Impedance Controller	Flexion	10	0.054	0.055	0.844	0.000	0.959
		30	0.219	0.219	0.965	0.041	0.985
		50	0.293	0.294	0.953	0.000	0.976
	Extension	10	0.145	0.146	0.961	0.000	0.987
		30	0.085	0.085	0.971	0.230	0.993
		50	0.341	0.345	0.939	0.000	0.974
	Supination	10	0.204	0.205	0.971	0.000	0.985
		30	0.804	0.808	0.579	0.000	0.860
		50	0.664	0.673	0.794	0.000	0.898
	Pronation	10	0.298	0.298	0.936	0.100	0.968
		30	0.338	0.338	0.943	0.000	0.974
		50	0.837	0.849	0.617	0.000	0.840
Sliding Mode Controller	Flexion	10	0.126	0.129	0.158	0.000	0.602
		30	0.212	0.212	0.968	0.002	0.990
		50	0.222	0.222	0.973	0.000	0.989
	Extension	10	0.193	0.196	0.931	0.000	0.966
		30	0.160	0.160	0.897	0.000	0.953
		50	0.223	0.225	0.974	0.000	0.987
	Supination	10	0.210	0.211	0.969	0.000	0.987
		30	0.236	0.240	0.963	0.000	0.982
		50	0.235	0.238	0.974	0.000	0.990
	Pronation	10	0.219	0.219	0.965	0.001	0.984
		30	0.224	0.224	0.975	0.020	0.988
		50	0.235	0.238	0.970	0.000	0.986

EST: Error Standard Deviation; RMSE: root mean square error; Corr: correlation.

## Data Availability

The datasets generated during and/or analyzed during the current study are available from the corresponding author upon reasonable request.
